# The Value of Applying Ethical Principles in Telehealth Practices: Systematic Review

**DOI:** 10.2196/25698

**Published:** 2021-03-30

**Authors:** Amanda Jane Keenan, George Tsourtos, Jennifer Tieman

**Affiliations:** 1 College of Medicine and Public Health Flinders University Bedford Park Australia

**Keywords:** telehealth, ethics, telemedicine, ethical, telecare, review, patient experience, care, effectiveness, framework

## Abstract

**Background:**

As the use of technology to deliver health services is increasing rapidly and has further intensified during the COVID-19 pandemic, these initiatives may fail if ethical impacts are not fully identified and acted upon by practitioners. Ignoring the ethical impacts of information and communication technology health service delivery creates an unintended risk for patients and can lead to reduced effectiveness, noncompliance, and harm, undermining the best intentions of governments and clinicians.

**Objective:**

Our aim was to explore how ethical considerations or impacts may be different, greater, or more variable in information and communication technology methods versus face-to-face health care delivery models, and how they may be applied in practice.

**Methods:**

We undertook a systemic literature review to provide a critical overview of existing research into the incorporation of ethical principles into telehealth practice. Six databases were searched between March 2016 to May 2016 and again in December 2020 to provide the benefit of currency. A combination of broad terms (“ethics,” “ethical,” “health,” and “care”) with the restrictive terms of “telehealth” and “telemedicine” was used in keyword searches. Thematic analysis and synthesis of each paper was conducted, aligned to the framework developed by Beauchamp and Childress.

**Results:**

From the 49 papers reviewed, authors identified or discussed the following ethical principles in relation to telehealth practice: autonomy (69% of authors, 34/49), professional–patient relationship (53% of authors, 26/49), nonmaleficence (41% of authors, 20/49), beneficence (39%, of authors, 19/49), and justice (39% of authors, 19/49).

**Conclusions:**

Although a small number of studies identified ethical issues associated with telehealth practice and discussed their potential impact on service quality and effectiveness, there is limited research on how ethical principles are incorporated into clinical practice. Several studies proposed frameworks, codes of conduct, or guidelines, but there was little discussion or evidence of how these recommendations are being used to improve ethical telehealth practice.

## Introduction

This literature review provides a critical overview of the existing research into the incorporation of ethical principles into telehealth practice because in the last decade, the use of information and communication technology (ICT) to deliver health services has rapidly grown into a rich tapestry of applications. Governments have sought to reduce health care expenditure and improve efficiency and access to care by the use of telephone, video, remote monitoring, or online methods [1]. Health service responses to the recent COVID-19 pandemic have further accelerated the use of telehealth globally, increasing the need for research and effective knowledge transfer mechanisms. Evaluations of telehealth programs have focused on the promises of telehealth, such as improved efficacy, efficiency, and clinical outcomes. Less research has focused on the potential perils of telehealth and the potentially negative, harmful, or unethical impacts of this type of service delivery [2]. The ethical considerations in ICT methods may be different, greater, or more variable as compared to face-to-face care models. The “major polarities of the medical practice,” including “respect for the patient, health-care quality and humaneness, as well as aiming at matching the needs of the whole population equitably” [3] are complicated by concerns about patient autonomy, the altered nature of the professional–patient relationship, the lack of the human touch in care, and the medicalization of the home environment [3,4]. Ignoring ethical impacts of ICT health service delivery creates unintended risk for patients and can lead to reduced effectiveness, noncompliance, and harm, undermining the best intentions of governments and clinicians [5,6].

For the purposes of this review the definition of telehealth is derived from the criteria outlined by the World Health Organization (WHO) [7]. This definition of telehealth includes the following: that the purpose of telehealth practice is to provide clinical support to patients by health care professionals, that it connects users who are not in the same physical location, that it involves the use of various types of ICT, and that its purpose is to improve health outcomes. The framework for the definitions, concepts and principles of health ethics is that provided by Beauchamp and Childress [8] and include respect for autonomy, nonmaleficence, beneficence, justice, and the professional–patient relationship*.*

## Methods

### Information Sources and Search Strategy

The sources of data included the Cochrane Database of Systematic Reviews, MEDLINE, Scopus, Web of Science, PubMed, and CINAHL databases were searched from March 2016 to May 2016, and again in December 2020 to provide the benefit of currency. The Cochrane database was included to obtain relevant control trials or clinical studies. MEDLINE, CINAHL, and PubMed were chosen as comprehensive databases of peer reviewed studies from the disciplines of medicine, nursing (particularity community nursing), and allied health professions (eg, psychology) that are most commonly associated with telehealth practice. Scopus and Web of Science were also included to supplement the results with studies from the social sciences and humanities, particularly philosophy and sociology, which had the potential to provide studies from an ethical, rather than a clinical or technological perspective. Studies using both qualitative and quantitative methods were included in the search criteria.

The terms used in the keyword search were “ethics,” “ethical,” “health,” “telehealth,” “telemedicine,” and “care”. During the search process the use of the broad terms, “ethics,” “ethical,” “health,” and “care,” was combined with the restrictive terms of “telehealth” and “telemedicine”.

### Grey Literature

Extended searching of internet sites, conference abstracts, and presentations was undertaken to identify any relevant grey literature that was not uncovered in the database search. Given the pilot or proof-of-concept structure of some telehealth services, relevant material might have been available in an unpublished form.

### Inclusion and Exclusion Criteria

The criteria provided by the WHO definition of telehealth was used to guide the inclusion and exclusion process at the first refinement stage.

#### Stage 1: Inclusion and Exclusion Criteria Used at the Abstract and Title screen

Eligible for inclusion and critical appraisal were studies that examined or discussed a relationship between health or medical ethics and the delivery of health services by connecting patients and providers in different physical locations using ICT with the purpose of improving health outcomes.

Ineligible for inclusion were studies relating to the ethical use of digital health service delivery methods that did not include an interaction with a health professional through telephonic methods, such as the use of sensors and assistive technologies in the home, or the use of mobile health apps. Studies that included both telehealth services and nontelehealth services together were included. Duplicate studies were also removed.

#### Stage 2: Inclusion/Exclusion Criteria Used at the Full-Text Screen

The final studies reviewed at the full-text screening stage were further required to satisfy the inclusion criteria of identifying or discussing ethical principles in relation to telehealth practice, ethical dilemmas or challenges in telehealth practice, or the ethical framework of telehealth practice.

## Results

**Figure 1:**
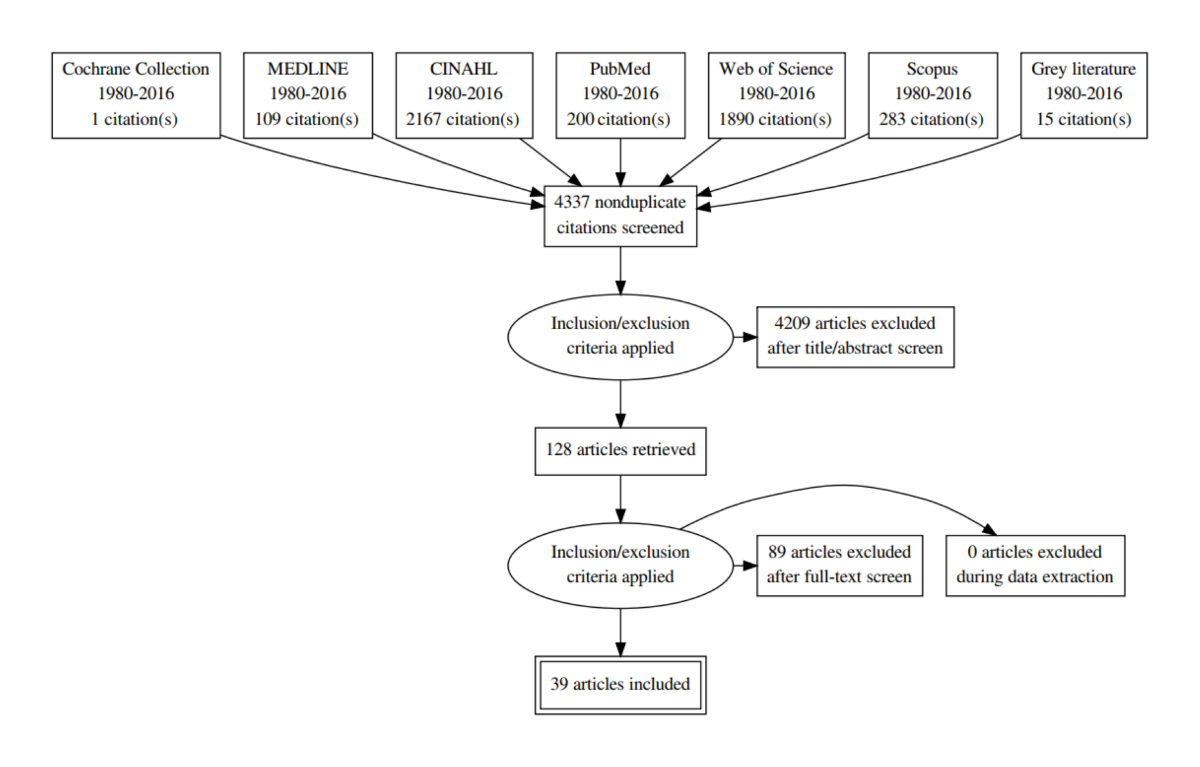
Flow of the citations reviewed as part of the systematic review 1980-2016.

Figure 1 illustrates the flow of the citations reviewed. The PRISMA (Preferred Reporting Items for Systematic Reviews) diagram indicates that most of the citation results came from CINAHL and the Web of Science databases. The content of these databases predominantly consists of research from the nursing, allied health, and social science disciplines, as well as grey literature. PubMed and MEDLINE produced fewer results, but these citations proved to be, in some cases, more relevant to the search terms, particularly the application of biomedical ethics in practice. The Cochrane database initial search produced only 1 citation of a reviewed control trial, and this was later excluded as it did not meet the second stage inclusion criteria, suggesting few clinical control trials have been undertaken regarding health ethics and telehealth practice.

**Figure 2:**
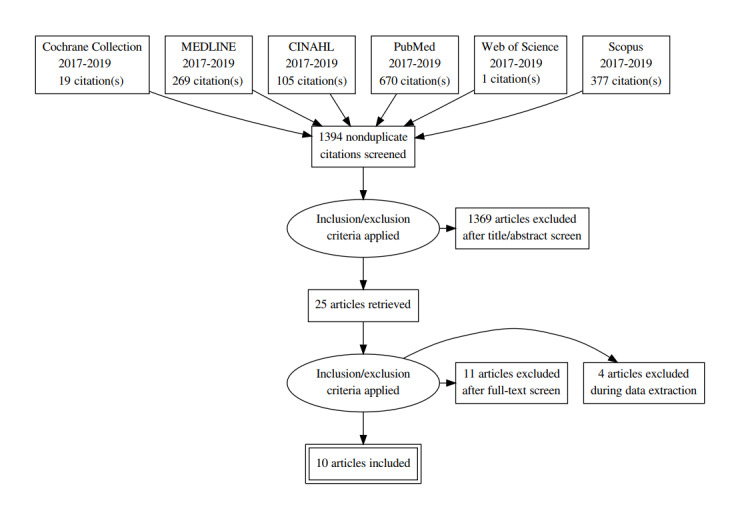
Flow of the citations reviewed as part of the systematic review 2017-2019.

The updated search, shown in Figure 2, produced 19 papers from The Cochrane database, suggesting the need for an increase in empirical evidence in this field. A summary of the ethical themes and distribution by article is shown in Table 1.

**Table 1:** Summary of themes and distribution of the discussion by article.

Ethical theme	Article by author and date
Autonomy	Botrugno 2019; Chaet et al 2017; Clark et al 2010; Cornford et al 2001; Demiris et al 2006; Draper et al 2013; Eccles 2010; Fisk et al 2014; Fleming 2009; Glueckauf et al 2018; Heintz et al 2015; Holmstrom et al 2007; Kaplan 2008; Korhonen 2015; Langarizadeh et al 2017; Layman 2003; Loute et al 2017; Magnusson 2003; Mort et al 2015; Nelson 2010; Nelson et al 2013; Nesher et al 2011; Newton 2014; Palm et al 2013; Parks 2016; Percival et al 2006; Perry et al 2010; Roman et al 1997; Rutenberg 2008; Sävenstedt et al 2006; Schermer 2009; Sethi et al 2012; Skar et al 2018; Sorell et al 2012; Stowe et al 2011
Beneficence	Chaet et al 2017; Clark et al 2010; Cornford et al 2002; Eccles 2010; Holmstrom et al 2008; Iserson 2000; Loute et al 2017; Magnusson 2003; Nelson et al 2013; Nesher et al 2011; Perry et al 2010; Roman et al 1997; Rutenberg 2008; Shea 2008; Skar et al 2018; Voerman et al 2017; Willems 2005;
Justice	Botrugno 2019; Chaet et al 2017; Clark et al 2010; Cornford et al 2001; Demiris et al 2009; Eccles 2010; Fleming 2009; Heintz et al 2015; Holmstrom et al2007; Humbyrd 2019; Langarizadeh et al 2017; Layman 2003; Loute et al 2017; Magnusson 2003; Nelson 2010; Nelson et al 2013; Palm et al 2013; Perry et al 2010; Skar et al 2018
Nonmaleficence	Chaet et al 2017; Clark et al 2010; Cornford et al 2001; Eccles 2010; Fleming 2009; Glueckauf et al 2018; Gogia et al 2016; Humbyrd 2019; Iserson 2000; Langarizadeh et al 2017; Loute et al 2017; Magnusson 2003; Nesher et al 2011; Perry et al 2010; Roman et al 1997; Rutenberg 2008; Sarhan 2009; Sävenstedt et al 2006; Skar et al 2018; Voerman et al 2017; Willems 2005
Professional–patient relationships	Botrugno 2019; Barina 2015; Chaet et al 2017; Cheshire 2017; Clark et al 2010; Demiris et al 2009; Draper et al 2013; Fleming 2009; Gogia et al 2016; Humbyrd 2019; Iserson 2000; Kluge 2011; Korhonen 2015; Langarizadeh et al 2017; Nelson 2010; Pols 2010; Roman et al 1997; Sävenstedt et al 2006; Skar et al 2018; Stanberry 2001; Stowe et al 2010; Voerman et al 2017; Wade et al 2012; Willems 2005

In all, 49 articles were included in the analysis stage and incorporated into a data extraction table (Multimedia Appendix 1). As the literature search did not identify any clinical studies and the number of original qualitative research studies was low at 8, a thematic analysis approach, searching across the data to “find repeated patterns of meaning” was applied [9]. The analysis and synthesis of each paper was conducted using both inductive and deductive reasoning. The papers were organized in accordance with the type of study involved and the ethical principles, frameworks, or evaluation processes identified or discussed in each one as relevant to the 5 principles of biomedical ethics. Also recorded in the data extraction table were any ethical subthemes that were present in addition to the core 5 under examination.

Among the 8 included studies that used qualitative methods to collect data, ethnographic, interview, and focus group methodologies were used. Furthermore, 6 of these studies involved patients or caregivers and nurses or other health professionals. The remaining papers were systematic reviews or research that incorporated existing literature. In addition, 6 studies recommended an ethical framework, code of conduct, or system of evaluation for the ethical provision of telehealth services. One-fifth of all papers included were from 2017 onward, indicating an increasing interest in telehealth ethics, even prior to the COVID-19 pandemic.

## Discussion

### Overview of Acquired Studies and Themes

The broad search strategy yielded 49 initial results, but analysis identified few studies that described how ethical considerations are or may be incorporated into telehealth practice, whether in the home, community, or medical environment. Although a small number of qualitative studies identified relevant ethical issues associated with telehealth practice and subsequently discussed their potential impact on service quality from the perspective of patients, caregivers, and health professionals, there is scant research on how ethical principles are incorporated into telehealth practice [10-18]. Several studies proposed ethical frameworks, codes of conduct, or guidelines for telehealth service delivery that may be applied or followed by health professionals, but they provided little discussion, evidence, or evaluation of how these recommendations are being used to establish or improve ethical telehealth practice [6,15,19-22].

### Autonomy

Autonomy was the predominant ethical principle discussed in the literature, with 69% (34/49) of the authors identifying or discussing it in relation to telehealth practice. Within this primary theme, several subthemes emerged including consent, individual choice, independence, empowerment, control, and self-determination [23-25]. Two qualitative studies in Sweden found that autonomy can be both improved and diminished through the use of telehealth by increasing the freedom for older persons to remain living in their own homes, while also potentially contributing to their isolation and “being made captive” in their homes [16]. This issue of telehealth seeking to improve autonomy but actually having the opposite effect was noted in a UK study which found that, while the introduction of telehealth as part of a home telecare service for older patients can “drastically improve their autonomy,” it may also lead to an increased reluctance to move out of the home environment for even a small amount of time and thus reduce independence [10].

A qualitative study of telenurses identified issues relating to gender-specific and cultural concerns affecting autonomy and independence specific to females accessing care [17]. A further Swedish study involving patients and families found accessing education, information, and support at a time convenient to patients could increase autonomy and a sense of independence [14].

Recommendations for maintaining or improving autonomy in telehealth practice recognize that the concepts of choice and independence are not simple, particularly for older or more vulnerable patients, and decisions about what improves autonomy “takes place in a complex and changing context” [26]. Heintz et al reduce the concept of autonomy to the patient’s ability to give informed consent or participate fully in decision making; meanwhile, Palm recommends an ethical assessment design comprising 5 questions relating to patient autonomy, including co-design, behavioral adjustments, understanding of the system and control under different usage scenarios, whether it enhances independence, and if so, whether this independence is desirable [19,27]. Although a reduction in autonomy may be unavoidable for some telehealth patients, particularly in older users who are more accepting of “traditional” health care models, wherever possible, the “loss should be minimised” [15,28]. Layman [29] notes that the methods of data collection, storage, and manipulation used with telehealth may threaten patient autonomy if it becomes the primary source of information, and recommends a “multipronged approach” in incorporating ethical principles into practice, including regulations, standards, codes of conduct, and codes of ethics. The implications for ethical telehealth practice from the perspective of autonomy then are that care should be taken to robustly assess the impact on patients from a number of standpoints to reduce the potential risk [30].

### Beneficence

Analysis revealed that 39% (19/49) of the papers identified or discussed the ethical principle of beneficence, or “being disposed to act for the benefit of others” [8] in relation to telehealth practice. These authors all noted that telehealth has the potential to benefit people by providing assurance, increasing an individual’s confidence in managing their health, and reducing the dependence on professional caregivers or family [20,31,32]. Improving access, quality of health care availability, and the continuity of care are additional examples of telehealth increasing beneficence [31], as is the ability of patients to be treated in familiar surroundings rather than hospitals [33]. Although Beauchamp and Childress [8] note that “obligations to confer benefits can be linked to the goal of morality itself” and are an “implicit assumption” in the actions of medical professionals, the principle of beneficence informs rather than determines or justifies other moral principles. Thus, an ethical telehealth practitioner is one who provides information that empowers patients to act in their own best interest, and the wide availability of the telephone system in the majority of countries offers a greater capacity for the patients to control their own care [22,34]. From the perspective of families and caregivers, Magnusson [14] found that the use of telehealth can deliver beneficence by providing them with “education, information and support which would directly help them in their individual caring situation”. In developing and implementing telehealth policies and guidelines then, it may enhance practice to be able to clearly articulate the benefits to both patients and providers in design and delivery, so that telehealth remains “a support system for well-defined needs and not be pushed as an engineering solution to health” [35].

### Nonmaleficence

Our analysis further revealed that 41% (20/49) of the papers identified or discussed the ethical principle of nonmaleficence, or preventing harm, in relation to telehealth practice. Examples of telehealth’s ability to actively promote safety were identified, including telephone or video lines left open for providers to check on a patient at regular intervals acting as a security guarantee against harm occurring in the home, or the mode of delivery lowering the risk in patient care because of the lack of physical proximity of the health care worker to the patient [14,22]. The potential for harm is more prevalent, however, and includes telehealth equipment such as videophones situated in the home having the effect of stigmatizing a person and causing shame or embarrassment, the possibility that professional caregivers may choose remote communication rather than delivering care in person in difficult or high needs cases may put clients at risk, and an “undue burden” [10] being imposed on unwell or frail patients who find the technology intrusive or do not fully understand its use [12]. A ethnographic study in the Netherlands with nurses and their patients found that “the feeling of safety and security the patients experienced, may not always have been realistic” due to nurses having to make value judgements about the types of information that were most important during telehealth sessions [11].

Sarhan [36] links confidentiality, nonmaleficence, and the professional responsibility of practitioners to ensure patients are protected from “emotional, spiritual, social or material” harm, while Willems [33] notes that using telehealth instead of traditional methods of health care may lead to families and caregivers being “loaded with more and different responsibilities”. Nesher [37] suggests that the additional layers of technology may compromise patient care by adding complexity and obscuring the most important information from clinicians. The responsibility to “respect, preserve and defend the patient’s dignity” has also been identified and linked to person-centered practice and user-driven design as core to ethical telehealth services [38]. A recent study of psychologist’s telebehavioral health practices noted that over half of the survey respondents reported “inadequate skills in managing crisis situations in the context of online practice,” including managing suicide risk [18]. The implications for practice here are that potential harms are not straightforward or easy to discern and may not be captured in established procedures or service evaluation tools.

### Justice

Results indicated that 39% (19/49) of papers identified or discussed the ethical principle of justice in relation to telehealth practice. Justice was most discussed in relation to fairness concerning equal access to telehealth technology balancing the needs of the individual with those of the wider community, ensuring not to disadvantage one group in favor of another [10]. Examples are given where the key advantage for providing telehealth—access to care for marginalized communities—is negated by the affordability of the technology or creates additional barriers for “at risk” patients [39,40]. In the case of mental health services, Nelson et al [41] note that the criteria set by mental health professionals of only using high-standard equipment can impact the ability of some localities to make telehealth services available, while Nesher [37] suggests that locations most likely to be in need of and benefit from telehealth services—rural areas—are likely to be least able to afford them. Perry et al [10] note the distinction between “individual level” and “system level” equity, arguing that benefits derived from the use of telehealth can positively impact in other areas of social care. Demiris [42] points out that providing underserved older adults access to services should not be done solely as a cost-saving exercise that “deprives patients of face-to-face consultations,” while Fleming [43] argues that special skills in telehealth delivery should be developed to ensure access for older patients in nursing homes—the “underserved”—and ethnic minorities. When considering justice in relation to developing a telehealth practice, questions related to equal access and fair distribution of the technology, and whether a digital or information divide exists should be used to guide the implementation of telehealth services in practice [27]. Models should be evaluated, not just in terms of resource allocation but also in relation to “the principle of human value” as well as any current legislation against discrimination [19]. Botrugno [44], in discussing the argument for telehealth to underpin greater distributive justice in health care, advises against accepting “technological determinism,” arguing instead for a “plan of analysis through which to critically assess the implications of telehealth” [44].

### Professional–Patient Relationships

Our review further found that 53% (26/49) of the papers identified or discussed the potential disruption of the relationship between health professionals and their patients, with several subthemes emerging including confidentiality, privacy, and fidelity [21,45-47]. The lack of the “human touch” in care has been identified as a key concern in providing health services remotely although the importance of this may vary between disciplines, such as teledermatology where it may be low and telepsychology where it may be much greater [42]. As more health services are delivered in “the virtual realm” rather than in physical proximity, the risk increases of “creating a distance between touch and care” [48]. Fleming [43] suggests that telehealth should not be used to replace the traditional face-to-face methods of health care delivery “that remains crucial to healing” but rather should be viewed as a supplementary method to improve care and treatment.

The undermining of trust between patients and their health care providers was discussed within the ethical subtheme of fidelity, with Chaet [49] asserting that the practice of medicine is “inherently a moral activity, founded in a covenant of trust between patient and physician,” which must be sustained. Trust and mutual respect may be challenged between patients and providers in a telehealth environment, particularly if the two have never met in person [50], as through “words and nonverbal actions the patient and the physician establish a relationship of trust that is essential to good medical care” [51]. The notion of not just trust but “sound trust” has been raised in relation to telehealth, whereby additional actions or behaviors are required by health professionals to win public trust “in the face of the conflicting interests that are at stake” [52]. Trust may also be undermined by the skepticism or caution generated by unfamiliar equipment being directed towards the health professional or by the reluctance of patients to speak freely in the presence of such equipment due to privacy or communication concerns [53,54]. Savenstedt [12] links the use of technology to the notion of “superficial care” arising from the “superficial” relationship created by the replacement of face-to-face care with remote care, but also notes that communicating through the use of telehealth may reduce loneliness in people who otherwise would have few options for interaction. Finally, Wade et al [13] found that patients may in some cases find a telehealth communication setting more protected and feel they are more likely to be listened to by the health provider, but also suggested that palliative care patients may suffer through a lower-quality therapeutic relationship, as may “groups or families”. The implication is that care should be taken around context and patient preferences for the relationship with telehealth practitioners when designing services.

### Limitations

Limitations noted during the implementation of the search strategy were the following: broad search terms such as “ethics” and “ethical” might have resulted in missing relevant papers that used similar but different terms, such as “moral,” “virtue/virtues,” or “values”; manual searches of journals specifically dedicated to studies on the use of ICT in health care might have provided additional suitable studies for inclusion; and several studies that were identified for inclusion at the first stage were not able to be obtained in their entirety and consequently could not be assessed for the second stage or included in the results.

### Conclusions

Our findings suggest that the principles of biomedical ethics are relevant to the context of telehealth practice and that interest in how ethical principles impact telehealth service delivery for patients and clinicians is increasing. We have identified a number of considerations for future telehealth policy and practice development to reduce the risk to patient experience and improve clinical care and delivery effectiveness and sustainability. Further research into how ethical principles are incorporated into organizational telehealth policies and models of care documentation would identify how ethical priorities are aligned with care delivery in current practice. Investigation and analysis of how ethical principles are incorporated into telehealth practice from both a patient and provider experience would identify gaps and opportunities to develop purposeful frameworks and guidelines, supported by an appropriate knowledge transfer model for telehealth clinicians.

## Appendix

Multimedia Appendix 1 Table 2 Data Extraction.

## Abbreviations

ICT: information and communication technology
PRISMA: Preferred Reporting Items for Systematic Reviews
WHO: World Health Organization
