# Supplementary material for: The Value of Applying Ethical Principles in Telehealth Practices: Systematic Review
Source: J Med Internet Res. 2021 Mar 30;23(3):e25698. doi: 10.2196/25698 (PMC8044738; doi:10.2196/25698)
Supplement: Multimedia Appendix 1 [file jmir_v23i3e25698_app1.doc]

**Appendix**

**Table 2 - Data Extraction**

| **Author/Year** | **Location** | **Study Type** | **Title** | **Ethical Themes** | **Sub Themes** | **Publication type** |
| --- | --- | --- | --- | --- | --- | --- |
| **Barina 2015** | USA | Research paper | New Places and Ethical Spaces: Philosophical Considerations for Health Care Ethics Outside of the Hospital | Professional-patient relationships | Ethics of care; communication | Peer reviewed journal |
| **Botrugno 2019** | USA | Research paper | Towards an Ethics for Telehealth | Autonomy; professional-patient relationships; justice | Confidentiality: privacy: need for new ethics, to address the operation of the remote services. | Peer reviewed journal |
| **Clark et al. 2010** | USA | Research paper | Telemedicine: Medical, legal and ethical perspectives | Autonomy; professional-patient relationships; beneficence; non-maleficence; justice | Confidentiality; privacy; respect for persons | Peer reviewed journal |
| **Chaet et al 2017** | USA | Research paper | Ethical Practice in Telehealth and Telemedicine | Autonomy; professional-patient relationships; justice | Trust; fidelity; differentials in access | Peer reviewed journal |
| **Cheshire 2017** | USA | Research paper | Telemedicine and the Ethics of Medical Care at a Distance | Professional-patient relationships | Limitations of perception; separation of action from effect; absence of personal presence | Peer reviewed journal |
| **Cornford et al. 2001** | UK | Research paper | Ethical Perspectives in Evaluation of Telehealth | Autonomy; beneficence; non-maleficence; justice | Ethical evaluation of telehealth services needs to be more extensive than face-to-face | Peer reviewed journal |
| **Demiris et al. 2009** | USA | Methodological Review | Ethical Considerations Regarding the Use of Technology for Older Adults: The Case of Telehealth | Justice; professional-patient relationships | Confidentiality; privacy; informed consent; equal access; usability | Peer reviewed journal |
| **Demiris et al. 2006** | USA | Research paper | Ethical Considerations for the Utilization of Telehealth Technologies in Home and Hospice Care by the Nursing Profession | Autonomy | Confidentiality; privacy; informed consent; equal access; usability; autonomy vs. dependence; medicalisation of the home environment; lack of human touch | Peer reviewed journal |
| **Draper et al. 2013** | USA | Research paper | Telecare, remote monitoring and care | Professional-patient relationships; autonomy | Empowerment | Peer reviewed journal |
| **Eccles 2010** | UK | Research paper | Ethical Considerations Around the Implementation of Telecare Technologies | Autonomy; beneficence; non-maleficence; justice | Ethics of care | Peer reviewed journal |
| **Fisk et al. 2013** | UK | Research paper | Telehealth and Service Delivery in the Home: Care, Support and the Importance of User Autonomy | Autonomy | User Autonomy | Book chapter |
| **Fleming 2009** | USA | Research paper | Telehealth Ethics | Autonomy; professional-patient relationships; non-maleficence; justice | Consent, privacy and confidentiality; justice, access and equity; burden and quality of life; exploitation | Peer reviewed journal |
| **Glueckauf et al. 2018** | USA | Quantitative study: A purpose sample of 164 Psychologists. The 28-item survey instrument focused on five key domains: providers’ use of telecommunication modalities; client population characteristics; professional, ethical, and legal/regulatory issues; and telehealth training and practice. | Survey of Psychologists’ Telebehavioral Health Practices: Technology Use, Ethical Issues, and Training Needs | Autonomy: non-maleficence | Consent: privacy: handling emergencies remotely | Peer reviewed journal |
| **Gogia et al. 2016** | USA | Research paper | Unintended Consequences of Tele Health and their Possible Solutions | Non-maleficence; professional-patient relationships | Miscommunication between providers as well as providers and patients | Peer reviewed Journal |
| **Heintz et al. 2015** | Sweden | Framework | Framework for Systematic Identification of Ethical Aspects of Healthcare Technologies: The SBU Approach. | Justice; autonomy | Equality and Justice; autonomy, privacy, cost-effectiveness | Peer reviewed journal |
| **Holmstrom et al.2007** | Sweden | Qualitative study: A purposeful sample of 12 female telenurses in Sweden was interviewed twice during 2004 and 2005. | The faceless encounter: ethical dilemmas in telephone nursing | Autonomy; beneficence; justice | Talking through a third party; confidentiality; insufficient resources | Peer reviewed journal |
| **Humbyrd 2019** | USA | Research paper | Virtue Ethics in a Value-driven World: Ethical Telemedicine | Non-maleficence; justice; professional-patient relationships | Virtue ethics; moral character | Peer reviewed journal |
| **Iserson 2000** | USA | Research paper | Telemedicine: A Proposal for an Ethical Code | Professional-patient relationships; beneficence; non-maleficence | Privacy; confidentiality | Peer reviewed journal |
| **Kaplan 2008** | USA | Research paper | Ethical Challenges of Telemedicine and Telehealth | Autonomy | Informed Consent; empowerment | Peer reviewed journal |
| **Kluge 2011** | Canada | Research paper | Ethical and legal challenges for health telematics in a global world: Telehealth and the technological imperative | Professional-patient relationships | Privacy; consent; misunderstanding of information; dependence on technology | Peer reviewed journal |
| **Korhonen 2015** | Finland | Systemic Literature Review | Technology and its ethics in nursing and caring journals: An integrative literature review | Autonomy; professional-patient relationships | Misunderstanding of information; beneficence; autonomy; fidelity; justice | Peer reviewed journal |
| **Layman 2003** | USA | Research paper | Health Informatics Ethical Issues | Autonomy; beneficence; justice | Beneficence | Peer reviewed journal |
| **Langarizadeh et al. 2017** | Iran | Systemic Literature Review | Application of Ethics for Providing Telemedicine Services and Information Technology | Autonomy: justice; professional-patient relationships | Beneficence | Peer reviewed journal |
| **Loute et al. 2017** | France | Research paper | What Ethics for Telemedicine? | Autonomy; professional-patient relationships; beneficence; non-maleficence; justice | Dehumanization of medicine; power; common good | Book chapter |
| **Magnusson 2003** | UK,  Ireland,  Sweden, Portugal | Qualitative study: Methods included interviews, focus groups and questionnaires with participating families, as well as professional carers and health and social care providers | Ethical issues arising from a research, technology and development project to support frail older people and their family carers at home | Autonomy; beneficence; non-maleficence; justice | Security; privacy; confidentiality | Peer reviewed journal |
| **Mort et al.2015** | UK,  Spain,  The Netherlands,  Norway | Ethnographic study (observation, work shadowing), interviews, older citizens’ panels and a participative conference. | Ethical implications of home telecare for older people: a framework derived from a multisited participative study | Autonomy | Privacy; intrusion | Peer reviewed journal |
| **Nelson 2010** | USA | Research paper | The Ethics of Telemedicine: Unique nature of virtual encounters call for special sensitivities | Autonomy; professional-patient relationships, justice | Informed consent; privacy; confidentiality; equity | Peer reviewed journal |
| **Nelson et al. 2013** | USA | Book chapter | Ethical Considerations in Providing Mental Health Services Over Videoteleconferencing | Beneficence; nonmaleficence; justice | Fidelity and responsibility; respect for rights and dignity, integrity | Book |
| **Nesher et al. 2011** | Israel | Research paper | Ethical issues in the development of tele-ICUs | Autonomy; beneficence; non-maleficence; justice | Autonomy | Peer reviewed journal |
| **Newton 2014** | USA | Research paper | The promise of telemedicine | Autonomy | Informed consent; access | Peer reviewed journal |
| **Palm et al. 2013** | Sweden | Research paper | Ethically sound technology? | Autonomy | Autonomy; privacy; freedom of choice; consent; human contact | Peer reviewed journal |
| **Parks 2015** | USA | Research paper | Home-Based Care, Technology, and the Maintenance of Selves | Beneficence; autonomy | Empowerment; identity; privacy; social isolation | Peer reviewed journal |
| **Percival et al. 2006** | UK | Qualitative Study: 22 focus groups, 5 with carers, 7 with professionals, and 10 with older people | Big brother or brave new world? Telecare and its implications for older people’s independence and social inclusion | Autonomy | Choice and self-determination; empowerment; independence; privacy | Peer reviewed journal |
| **Perry et al. 2010** | UK | Delphi study developed with patients for provider respondents n=23 | Ethical issues in the use of telecare | Autonomy; beneficence; non-maleficence; justice | Privacy; isolation and wellbeing; fairness in the allocation of resources | Grey Literature  (report) |
| **Pols 2010** | The Netherlands | Ethnographic study: Observation and interviews with 9 specialised nurses and 33 patients. | The Heart of the Matter. About Good Nursing and Telecare | Professional-patient relationships | New types of communication and information from patients to nurses | Peer reviewed journal |
| **Roman et al. 1997** | USA | Research paper | Creating an ethical foundation for home telemedicine | Autonomy; beneficence; non-maleficence; justice; professional-patient relationships | Privacy; confidentiality | Peer reviewed journal |
| **Rutenberg 2008** | US | Research paper | Ethics in Telehealth Nursing Practice | Autonomy; beneficence; non-maleficence | Autonomy | Peer reviewed journal |
| **Sarhan 2009** | USA | Research paper | Telemedicine in healthcare 2: the legal and ethical aspects of using new technology | Non-maleficence | Confidentiality; privacy | Peer reviewed journal |
| **Sävenstedt et al. 2006** | Sweden | Qualitative study: An interview study with 10 healthcare personnel | The duality in using information and communication technology in elder care | Autonomy; non-maleficence; professional-patient relationships | Superficiality and genuineness; capacity and freedom; intrusion and privacy | Peer reviewed journal |
| **Schermer 2009** | The Netherlands | Research paper | Telecare and self-management: opportunity to change the paradigm? | Autonomy | Consent; privacy; equal access; medicalisation of the home environment | Peer reviewed journal |
| **Sethi et al. 2012** | UK | Research paper | Telecare: Legal, Ethical and Socioeconomic Factors | Autonomy | Consent | Peer reviewed journal |
| **Shea 2008** | USA | Research paper | Trust in the Virtual Home Healthcare Nurse | Beneficence | Trust | Peer reviewed journal |
| **Skar et al. 2018** | Sweden | Systemic Literature Review | The importance of ethical aspects when implementing eHealth services in healthcare: A discussion paper | Autonomy; beneficence; non-maleficence; justice; professional-patient relationships | Privacy; confidentiality; user-centred design | Peer reviewed journal |
| **Sorell et al 2012** | UK | Research paper | Telecare, Surveillance, and the Welfare State | Autonomy | Independence; privacy; isolation | Peer reviewed journal |
| **Stanberry 2001** | UK | Research paper | Telemedicine: barriers and opportunities in the 21st century | Professional-patient relationships | Privacy; confidentiality | Peer reviewed journal |
| **Stowe et al. 2010** | UK | Research paper | Telecare, telehealth and telemedicine | Autonomy; Professional-patient relationships; | Confidentiality; lack of face-to-face contact | Peer reviewed journal |
| **Velasquezb 2012** | USA | Research paper | Ethical Considerations in Providing Mental Health Services Over Video teleconferencing | Beneficence; non-maleficence; justice | Fidelity and responsibility; respect for rights and dignity, integrity | Peer reviewed journal |
| **Voerman et al. 2017** | USA | Research paper | Sound Trust and the Ethics of Telecare | Beneficence; non-maleficence; professional-patient relationships | Trust; sound trust | Peer reviewed journal |
| **Wade et al. 2012** | Australia | Qualitative study – 37 telehealth clinicians and managers were interviewed. | A qualitative study of ethical, medico-legal and clinical governance matters in Australian telehealth services | Professional-patient relationships | Privacy and security; consent and choice; empowerment; access | Peer reviewed journal |
| **Willems 2005** | The Netherlands | Research paper | Advanced home care technology: moral questions associated with an ethical ideal | Beneficence; non-maleficence; professional-patient relationships | Quality of communication; lack of face-to-face contact | Grey Literature  (report) |
